# Genotyping of *Haliotis discus hannai* and machine learning models to predict the heat resistant phenotype based on genotype

**DOI:** 10.3389/fgene.2023.1151427

**Published:** 2023-03-31

**Authors:** Eun Soo Noh, Sathiyamoorty Subramaniyam, Sunghyun Cho, Young-Ok Kim, Choul-Ji Park, Jeong-Ho Lee, Bo-Hye Nam, Younhee Shin

**Affiliations:** ^1^ Biotechnology Research Division, National Institute of Fisheries Science, Geoje, Republic of Korea; ^2^ Research and Development Center, Yongin-si, Gyeonggi-do, Republic of Korea; ^3^ Fisheries Seed and Breeding Research Institute, National Institute of Fisheries Science, Busan, Republic of Korea; ^4^ Fish Genetics and Breeding Research Center, National Institute of Fisheries Science, Geoje, Republic of Korea

**Keywords:** abalone, Haliotis discus hannai, heat resistant, genotype, aquaculture

## Introduction

Abalone (*Haliotis discus hannai*) is an expensive seafood in Asian countries and an important species in fishery and mariculture industries, generating marginal revenue for the Chinese and South Korean economies. South Korea is the second largest producer of abalones, followed by China (Nam et al., 2016). Thus far, approximately 70 species of *Haliotis* have been discovered worldwide; among them, 7 have commercial importance and 6 are naturally distributed in South Korea (i.e., *Haliotis discus hannai*, *Haliotis discus*, *Haliotis madaka*, *Haliotis gigantea*, *Haliotis diversicolor*, and *Haliotis diversicolor supertexta*) (Adachi and Okumura, 2012). In particular, the species *Haliotis discus hannai* is farmed widely in coastal regions of South Korea (Im and Kim, 2020). Two major factors affect abalone production in the sea: overfishing, due to its high market value, and increased atmospheric CO_2_, resulting in rising sea temperatures (Peter, 2016). While overfishing can be addressed by imposing strict laws and establishing a mariculture system, mitigating the changes in sea temperature is more difficult. Moreover, temperature fluctuations in sea water cause high mortality in marine cage-based abalone cultivation, particularly in the summer in coastal regions of South Korea (Kang et al., 2019). Thus, genetic/genome-assisted breeding could be a reasonable solution to increase abalone production in natural sea and mariculture systems. A draft reference genome of abalone is available, which could help determine genotypes for specific phenotypic traits (Nam et al., 2017). Many scientific reports have addressed the establishment of molecular datasets related to the physiological process of heat resistant traits, i.e., transcriptome (Tripp-Valdez et al., 2019; Kyeong et al., 2020; Kim et al., 2021), proteome (Kang et al., 2019), and metabolome (Xu et al., 2020) analyses. These datasets can be used to elucidate preliminary gene markers such as heat shock proteins (HSPs), which function in transcription and translation in abalone (Kyeong et al., 2020). Heat stress also alters energy metabolism and increases susceptibility to various pathogens, such as *Vibrio parahemolyticus* (Nam et al., 2016; Crosson et al., 2020), affecting the reproduction and growth of abalones (Swezey et al., 2020) as well as the metabolic rate in the digestive tract (Frederick et al., 2022). To our knowledge, no genotyping studies or datasets are available for *Haliotis discus hannai* other than a population assessment (Nam et al., 2021). In this study, we used genotyping-by-sequencing (GBS) to observe the genotypes associated with heat stress, and establish genotypic chip and machine learning (ML)-based prediction models to predict heat-sensitive abalones for breeding purposes with 96 single-nucleotide polymorphisms (SNPs).

## Significance of the data

Genotype data was generated for heat-resistant and -sensitive abalones from three different populations. Initially, 96 SNPs selected from the GBS dataset and those used to build the targeted Fluidigm chip were used for validation in two additional populations. These datasets were subjected to ML methods to develop a predictive model for heat-resistant and -sensitive abalone phenotypes. This dataset could be used to generate a genetic library for genome-assisted breeding for abalone.

## Materials and methods

### Experimental design for GBS and the fluidigm

**FIGURE 1:**
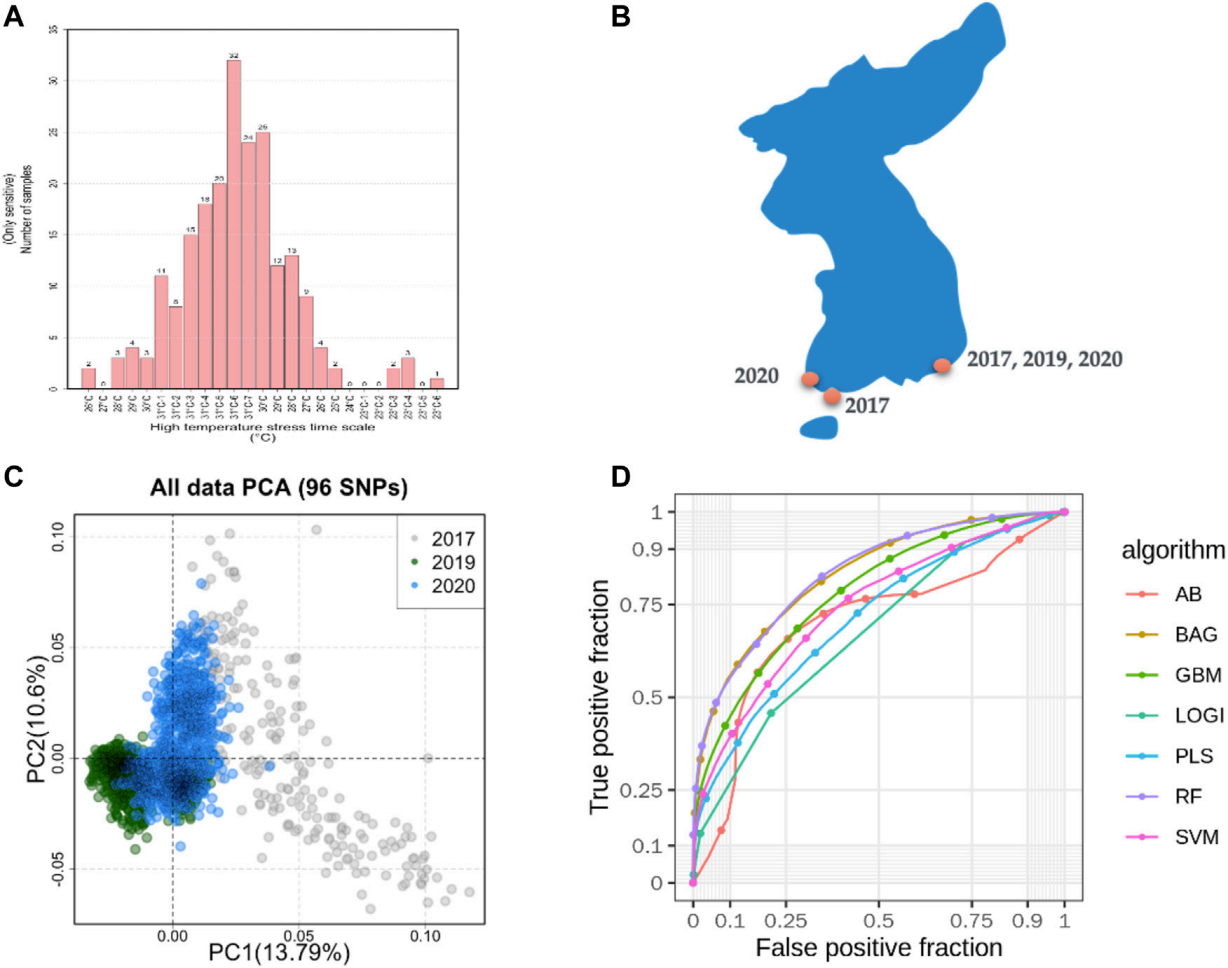
Sampling of machine learning predicted summaries. **(A)**. Heat resistance experiment for sample selection and defining the class as case and control. **(B)**. Sampling locations in South Korea. **(C)**. Sample diversity observed with 96 SNPs based on principal component analysis (PCA). **(D)**. Machine learning models predicted the accuracy from the pooled dataset.

A total of 400 abalones were selected from the Genetic and Breeding Research Center, NIFS, in Geoje, South Korea, and from a commercial farm in Wando and Heanam, South Korea (Supplementary Figure S1). The average shell length was 54.69 ± 3.78 mm, the shell width was 37.09 ± 2.63 mm, and the body weight was 16.44 ± 3.87 g. The experiment lasted 20 days (23 September 2016, to 12 October 2016). The abalones were maintained in a tank (1.2 × 3 × 0.8 m) with a constant flow of seawater at the Genetic and Breeding Research Center. The temperature was maintained at ≤ 24°C for the first 7 days to acclimate. The temperature was increased by 1°C per day for the next 7 days, until reaching a maximum temperature of 31°C, using an Aquatron system (Yoowon Electronics, Seoul, South Korea). During this time, the dissolved oxygen level was maintained at 7.8 ± 0.5 mg/L. The temperature was maintained at 31°C for an additional 6 days, during which the abalone mortality rate of the general population was 50%. Dead abalones were collected immediately for sampling. Foot muscles were collected and stored in ethanol. The heat resistant of the abalones was measured by survival time. The animals were categorized into two groups: heat-resistant and heat-sensitive (Figure 1A). Finally, 156 heat-resistant and 107 heat-senstive abalones were randomly selected from F4 population for sequencing. A similar experimental procedure was carried out for another two populations in 2019 and 2020 as shown in Table 1. In total, 2,282 samples were included from all three studies (Table 1; Supplementary Table S3).

**TABLE 1:** Selected SNP-assisted machine learning based prediction summary from three different combinations of datasets.

Dataset	GBS 2017	Fluidigm 2019	Fluidigm 2020	Balanced accuracy (96 SNPs)	Balanced accuracy (38 SNPs)
Size	107 controls	346 controls	440 controls		
	156 cases	806 cases	417 cases		
Type 3	Training and testing [70% (train and test), 30% independent dataset]			0.697	0.680
Type 2	Training and testing		Validation	0.486	0.463
Type 1	Training and testing	Validation		0.502	0.524

### GBS library preparation and sequencing

Total genomic DNA from the 263 samples was extracted from muscle tissue, quantified, and normalized to 20 ng/μL. The DNA (200 ng) was digested with 8 U of high-fidelity *Pst*I at 37°C for 2 h and heated to 65°C for 20 min to inactivate the enzyme. Six DNA libraries were constructed for GBS as described previously (Nam et al., 2021), pooled, and amplified by multiplex polymerase chain reaction (PCR). The products were purified using a QIAquick PCR Purification Kit (Qiagen, Hilden, Germany) and the distribution of fragment sizes was evaluated with a BioAnalyzer 2,100 instrument (Agilent Technologies, Santa Clara, CA, United States). The GBS libraries were sequenced with the Illumina NextSeq500 platform (San Diego, CA, United States) using 150-bp single reads in DNA Link, the authorized service provider. Library preparation has been described previously (Nam et al., 2021). A summary is provided in Supplementary Table S1.

### Variant calling, SNP selection, and estimate association

Sequences were subjected to quality and adapter trimming with Trimmomatic 0.32 using the following parameter settings: leading, 5; trailing, 5; sliding window, 4:15; and min, 30 (Bolger et al., 2014). The processed reads were mapped to the abalone reference genome (Nam et al., 2017) using Bowtie2 v.2.2.8 (Langmead and Salzberg, 2012) and variant calling was performed with the Haplotype caller in the Genome Analysis Toolkit (GATK) (McKenna et al., 2010). SNPs were selected with GATK parameters, i.e., normalized quality score ≥2 and mapping quality ≥40. Additionally, the missing genotypes were input with Beagle method v.4.1 (Browning et al., 2021). SNPs were annotated with SnpEff v.4.2 (Cingolani et al., 2012). Finally, high-quality SNPs were selected with the following steps: 1) bi-allelic sites, 2) genotyping rate of the samples at each variable site ≥90%, 3) minor allele frequency (MAF) > 5%, and 4) Hardy-Weinberg equilibrium (HWE) < 0.001 using PLINK1.9 (Purcell et al., 2007). The selected SNPs were subjected to population stratification with the STRUCTURE algorithm (Pritchard et al., 2000) with a K range of 1–7 with 10,000 iterations. The association between genotype and phenotype was estimated as follows. The samples were classified as heat-resistant (Case) or -sensitive (Control). Dead abalones were considered sensitive and the rest were considered heat-resistant. Features such as fixation index (Fst) and genomic nucleotide diversity (*π*) were calculated with VCFtools v. 0.1.3 (Danecek et al., 2011). Reduction of diversity (ROD) was determined by the following equation [ROD = 1 − (π of case/π of control)] within a 10 kb window size. The region at which the ROD >0.8 was defined as the selective sweep. The SNP ran from −5 kb to +5 kb in the gene region until the end of the gene. SNPs were considered to be significantly associated with the trait when *p* < 0.01; analyses were done using PLINK with the—assoc function.

### Targeted fluidigm chip design

A Fluidigm chip was designed with 96 SNP markers from the GBS dataset-assisted genome wide association study (GWAS). The target SNP genotyping chip was constructed with a Fluidigm 96.96 dynamic array integrated fluidic circuit (IFC) using an Adventa sample ID genotyping panel. For chip design, the primers for each SNP were selected as 100 bp of the flanking regions. Primers such as allele-specific primers (ASPs), locus-specific primers (LSPs), and specific target amplification primers (STAs) were designed using the Fluidigm SNP type assay protocol. The PCR cocktail was prepared with ASPs, LSPs, and STAs according to the manufacturer’s protocol along with the high-quality DNA prepared from each sample. The samples were loaded in a 96-well plate (12 columns x 8 rows) and subjected to the SNPtype 96X96 thermal cycling protocol with a FC1 PCR cycler to detect fluorescence by Biomark HD and processed with the SNP Trace™ Panel Analysis tool in the SNP genotyping analysis software. Genomic DNA (gDNA) quality was assessed by the concentration (ng/μLS) of each sample, which was measured with a Biotek Epoch spectrometer at 260/280 nm. All experimental protocols were performed by the TNT research service provider in Anyang, South Korea. A detailed summary is provided in Supplementary Table S2.

### Machine learning approach to predict phenotype

To assess the classification potential of selected SNPs, 7 ML models were used: AdaBoost (AB), Bagged Tree (BT), Generalized Boosted Regression (GBR), Boosted Logistic Regression (BLR), partial least squares (PLS), Random Forest (RF), and support vector machine with Linear Kernel (SVM-LK). In this study, we generated three datasets (datasets 1–3). We developed the basic models using a pooled dataset with individual validation datasets to understand the association of SNPs with three different populations. ML was performed using the ‘train’ in function in the caret package in R software with tenfold cross validation (ver. 3.3; R Development Core Team, Vienna, Austria) (Zhao, 2014; Emir et al., 2016). Model assessments were performed using parameters such as accuracy, kappa, sensitivity, specificity, pro pred value, negative pred value, precision, recall, F1, prevalence, and balance accuracy as described previously (Malik et al., 2022).

### Preliminary analysis report

In total, 185.9 GB of sequence was generated by GBS from 263 samples, and 81.59% of the reads were mapped to the abalone genome (Supplementary Figure S2). The mapped reads covered approximately 3% of the genome. From the mapped reads, 16,119 high-quality SNPs were obtained from 232,231 called SNPs, as illustrated in Supplementary Figure S3. Among those, 96 SNPs were selected with the metrics described in the Materials and Methods. In summary, 18 markers were selected using the ROD, 32 SNP markers were selected using the ODD score, and 46 markers were selected from the GWAS. The majority of the selected SNPs were present in intergenic regions and upstream of coding regions (Supplementary Figure S4). These SNPs were encoded with a Fluidigm chip for genotyping, and genotypes were generated from two other populations (Figure 1B) from different regions of Korea to include abalone diversity (Figure 1C). Detailed genotype summaries are provided in Supplementary Table S3. Finally, the 96 selected SNPs were subjected to the ML models to determine the predictive potential from. In this study, we used 7 ML methods (AB, BT, GBR, BLR, PLS, RF, and SVM-LK) with three combinations of genotyped datasets (Supplementary Table S4; Supplementary Figure S5, S6). We observed that an increase in the size of the dataset from different populations increased the ML prediction balanced accuracy (Table 1). Furthermore, the RF performed well in a pooled dataset (i.e., Type 3) with 0.714 balanced accuracy. Further, while optimizing the machine with 96 SNPs as features, we identified a subset of the SNPs (i.e., 38 SNPs) that contributed to the higher accuracy (Table 1; Supplementary Table S5). The features were selected with the same seven machines and the final features were selected from the seven machines with mean probabilities ≥0.1. This preliminary dataset could be a valuable asset to gain insight into heat resistance trait selection during abalone breeding. Detailed annotations of the SNPs are provided in Supplementary Table S5.

## Data Availability

The datasets presented in this study can be found in online repositories. The names of the repository/repositories and accession number(s) can be found in the article/Supplementary Material.
