# Supplementary material for: Genotyping of Haliotis discus hannai and machine learning models to predict the heat resistant phenotype based on genotype
Source: Front Genet. 2023 Mar 31;14:1151427. doi: 10.3389/fgene.2023.1151427 (PMC10102348; doi:10.3389/fgene.2023.1151427)
Supplement: Supplementary file 2 [file Image1.pdf]

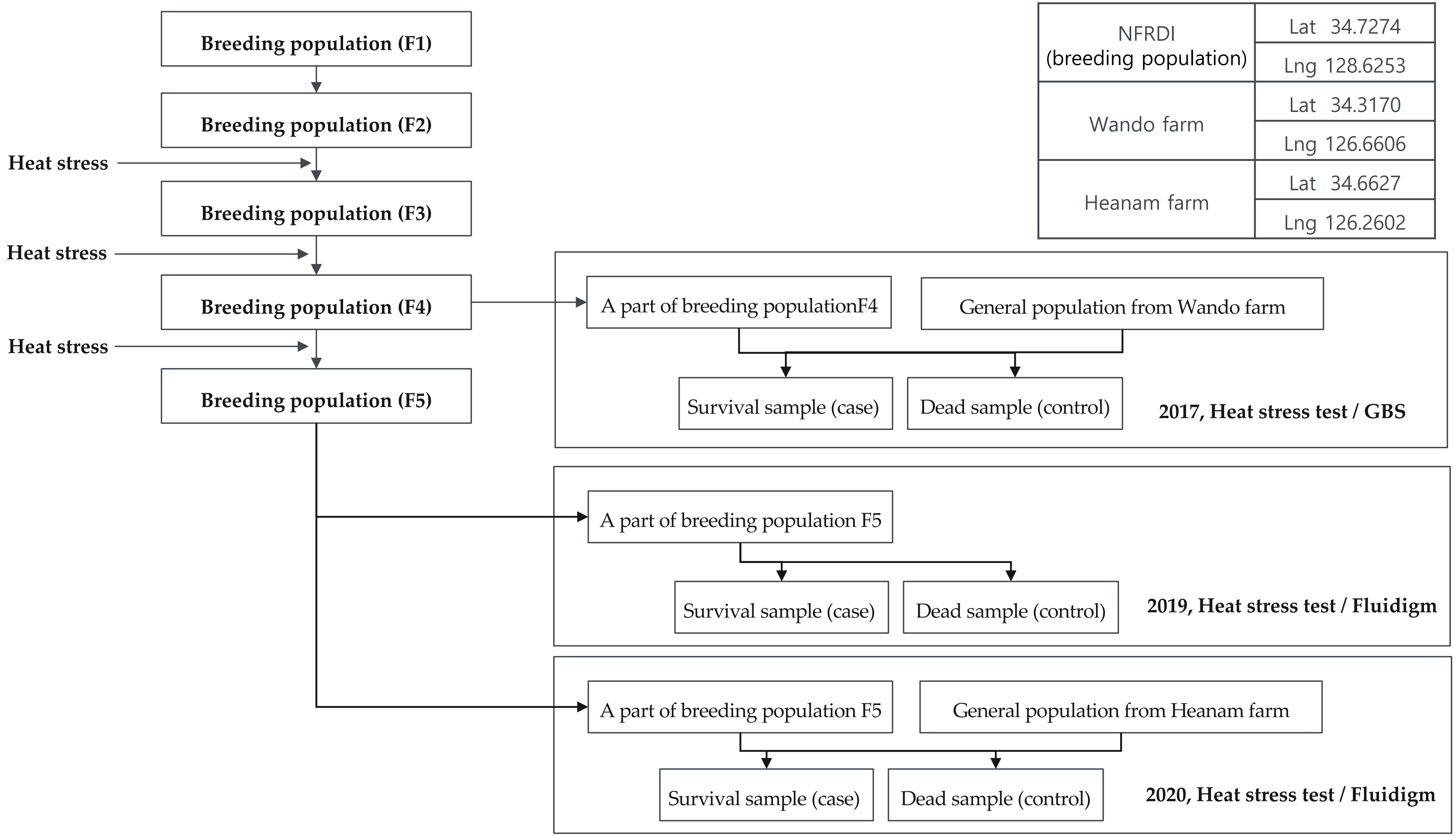

Supplementary Figure 1: Detail summary for the Abalone samplings.

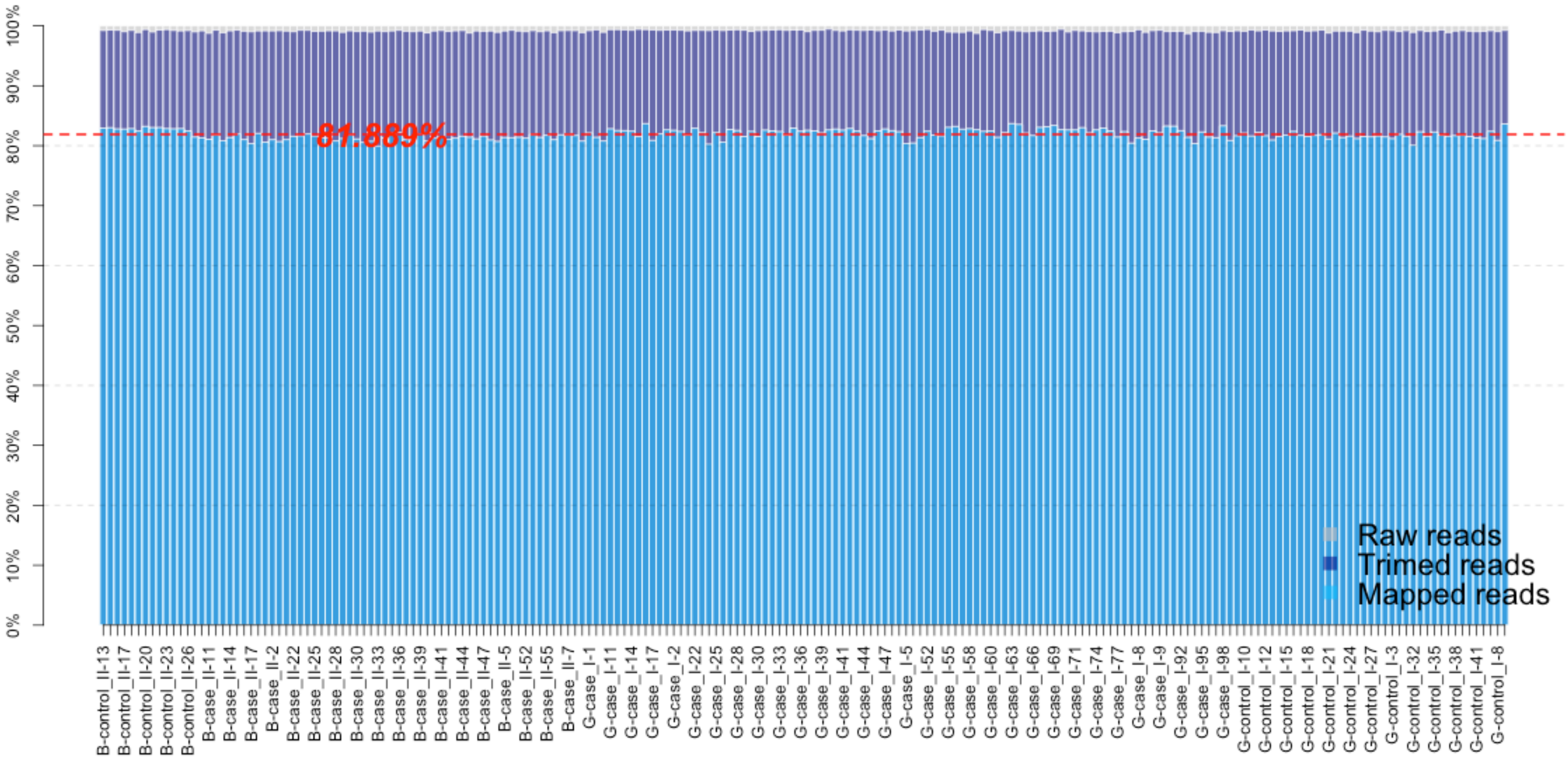

Supplementary Figure 2: GBS sequencing, preprocessing and summary of reference mapping to Abalone genome.

© 2022 INSILICOGEN, INC. ALL RIGHTS RESERVED.

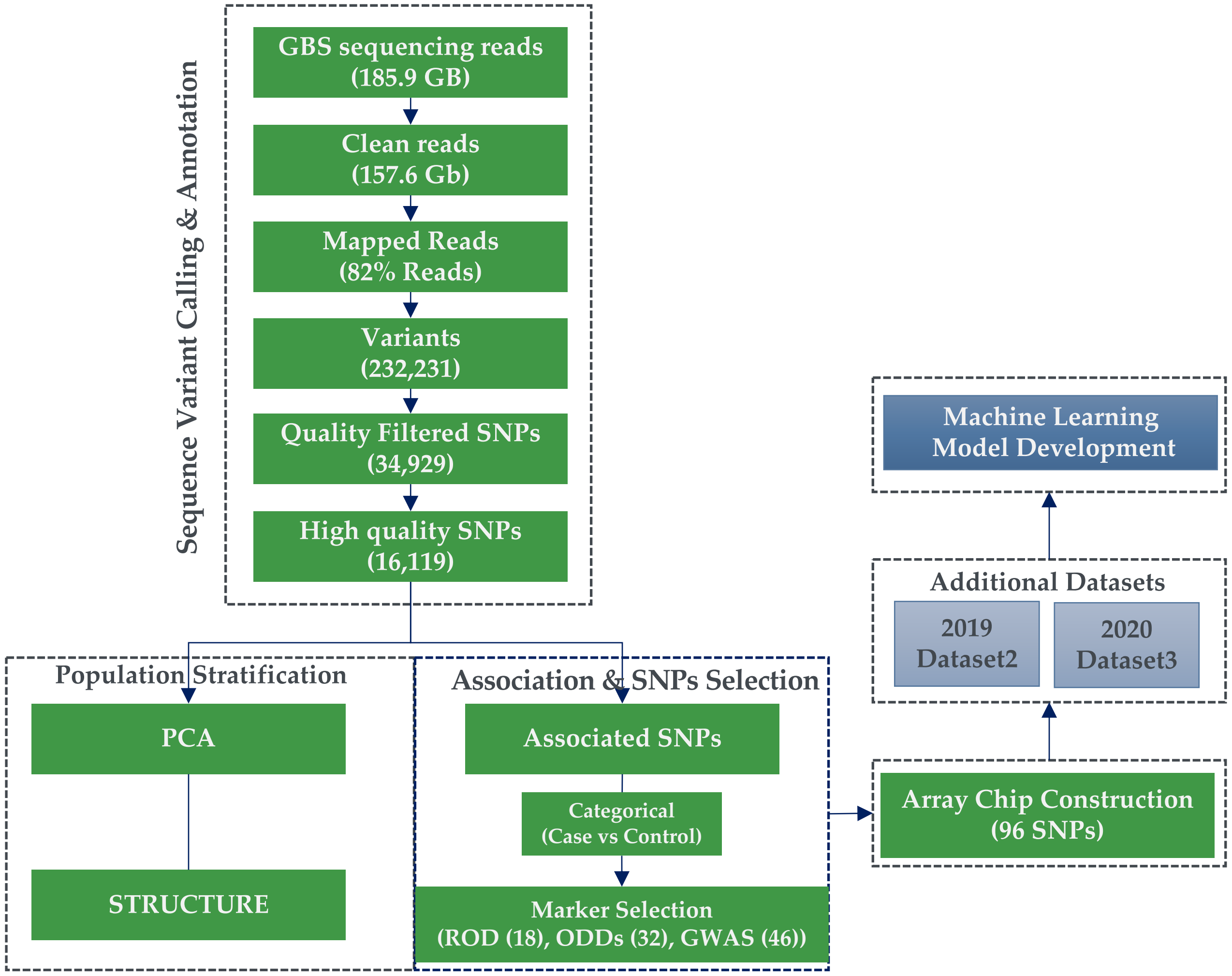

Supplementary Figure 3: Complete overview of the analysis workflow, which followed in this study to obtain the 96 SNPs and to construct the *Haliotis hannai* heat resistance classification machine models.

© 2022 INSILICOGEN, INC. ALL RIGHTS RESERVED.

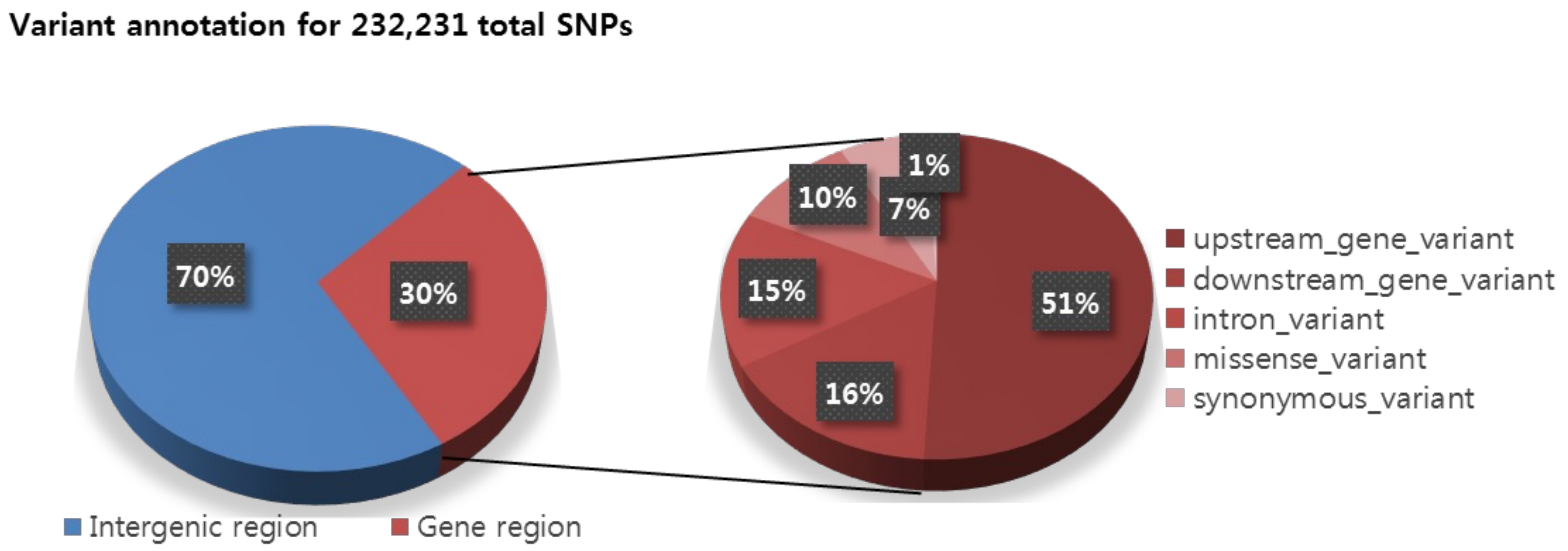

Supplementary Figure 4: GBS sequencing, preprocessing and summary of reference mapping to Abalone genome.

© 2022 INSILICOGEN, INC. ALL RIGHTS RESERVED.

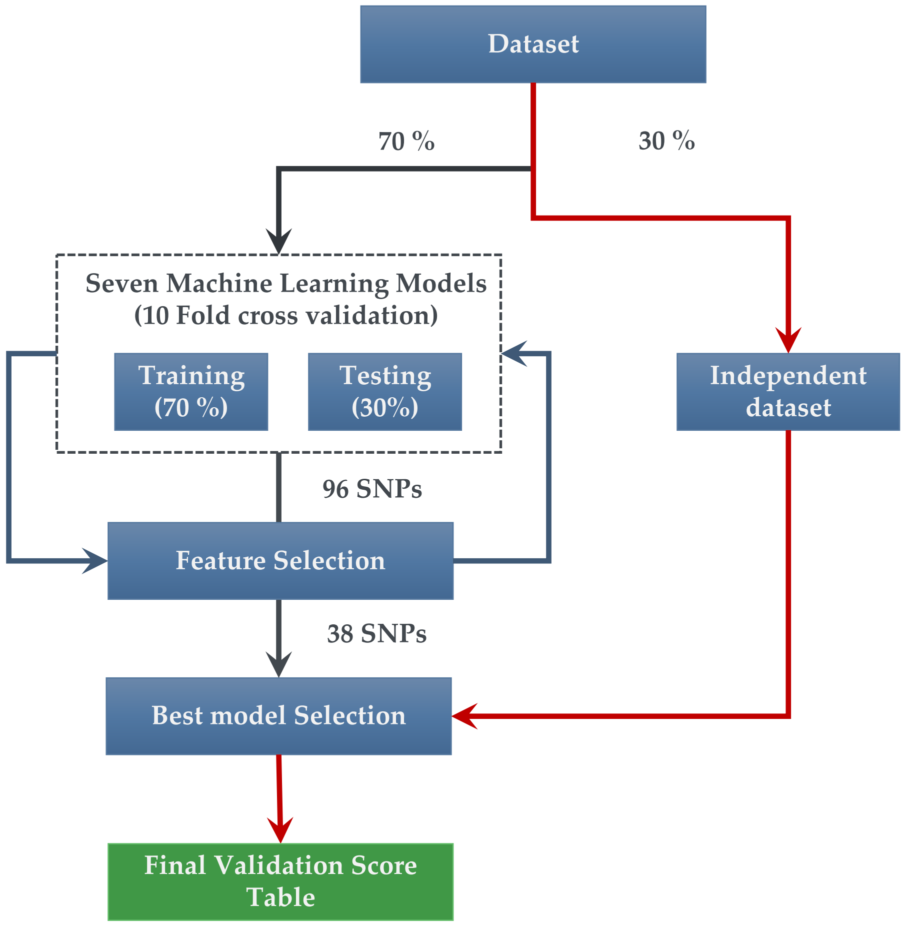

Supplementary Figure 5: Data set classifications for Machine Learning training, feature selection and validation process.

ⓒ 2022 INSILICOGEN, INC. ALL RIGHTS RESERVED.

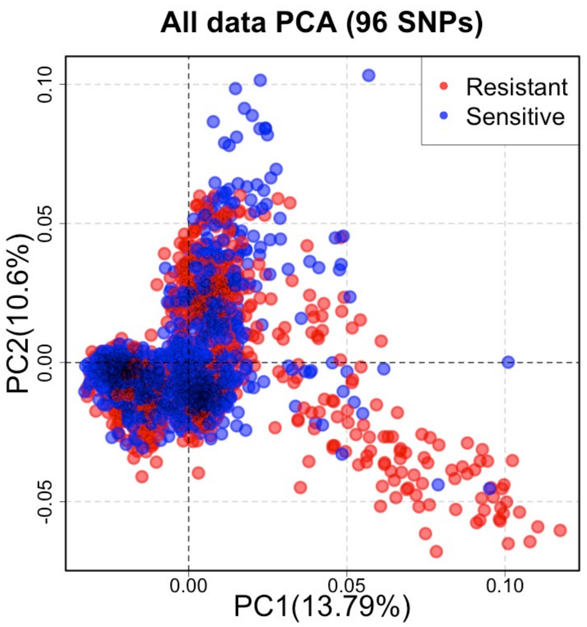

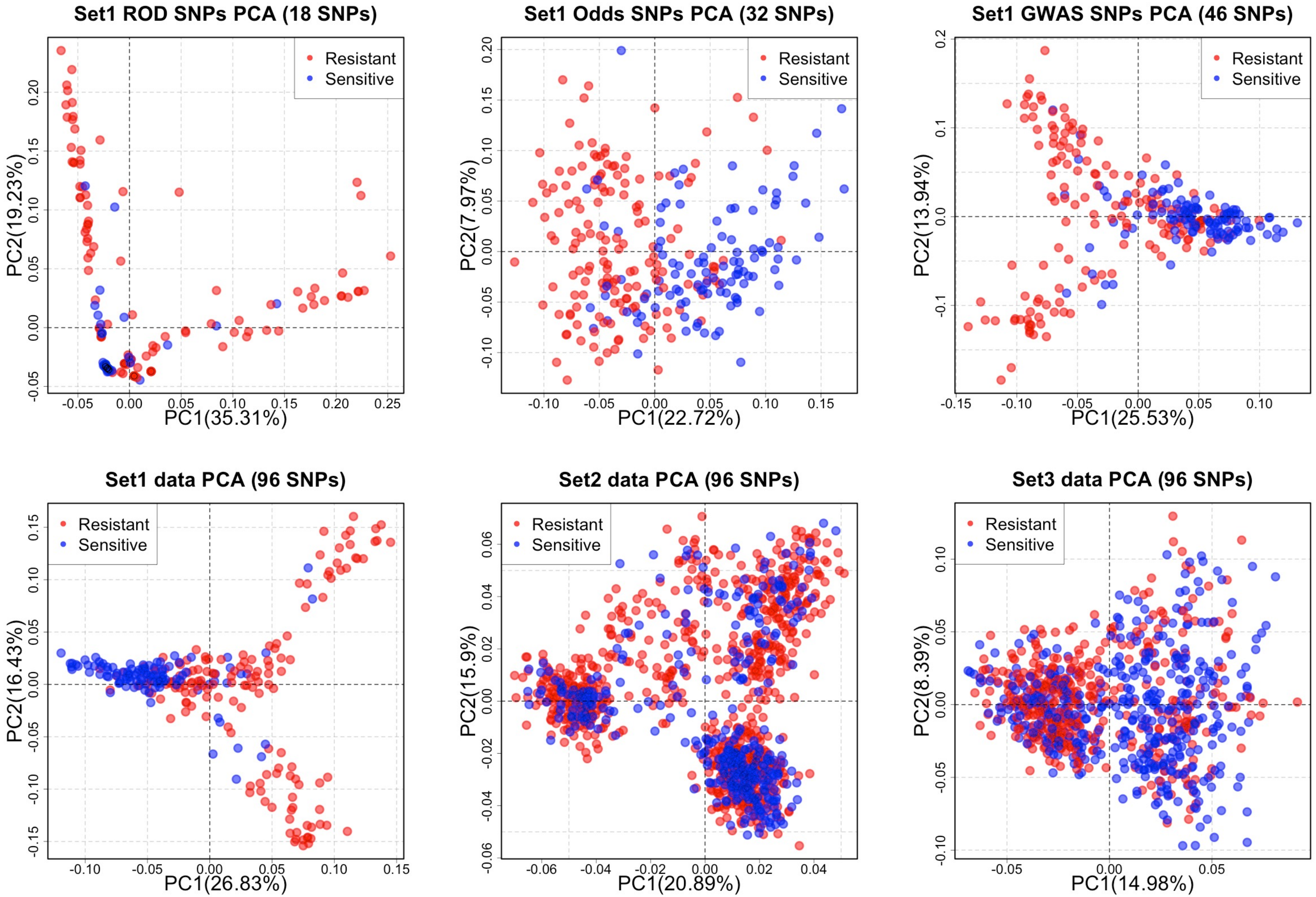

Supplementary Figure 6: Data set classifications for Machine Learning training, feature selection and validation process.

© 2022 INSILICOGEN, INC. ALL RIGHTS RESERVED.
